# Intimate partner violence: The need for an alternative primary preventive approach in Botswana

**DOI:** 10.4102/phcfm.v10i1.1699

**Published:** 2018-05-24

**Authors:** Radiance M. Ogundipe, Nataly Woollett, Gboyega Ogunbanjo, Anthony A. Olashore, Stephane Tshitenge

**Affiliations:** 1 Department of Family Medicine and Public Health, University of Botswana, Botswana; 2 School of Public Health, University of the Witwatersrand, South Africa; 3 Department of Family Medicine and Primary Health Care, Sefako Makgatho Health Sciences University, South Africa; 4 Department of Psychiatry, Faculty of Medicine, University of Botswana, Botswana

## Abstract

Intimate partner violence is a common social problem which causes considerable relationship stress and results in significant morbidity and mortality of the victims. Botswana, like many other countries in sub-Saharan Africa, has tried to address the problem of intimate partner violence with legislations prescribing punitive measures for the perpetrators and protection for the victims. The effectiveness of these measures in reducing the prevalence of intimate partner violence is doubtful. This article is to motivate for an alternative primary preventive approach to the problem as a more pragmatic option.

## Introduction

Intimate partner violence (IPV) is a social problem with global prevalence. It is described as physical, emotional, stalking, sexual or psychological oppression of an individual by a person who is privy to the individual’s life by virtue of a current or past intimate relationship.^1,2^ An intimate partner usually has regular contact and emotional connectedness with the individual. They are often familiar with the individual and may be recognised socially as couples. However, sexual intimacy is not compulsory to define an intimate partner relationship.^1,3^ Chapter 2 of Section 7 of the Constitution of Botswana adopted in 1966, and amended in 2006, includes the following provision: No person shall be subjected to torture or to inhuman or degrading punishment or other treatment.^4^ By implication, IPV falls within the ambit of this provision. The *Domestic Violence Act* 2008 of Botswana specifically describes actions that constitute IPV and prescribes legal steps that can be resorted to by the victims for their protection and enforcement of their rights as enshrined in the constitution.^5^ It is doubtful if these legal measures have made any impact on the perpetration of IPV in Botswana. There are no consistent structured measures applied to prevent IPV in Botswana as most victims of IPV prefer keeping the problem to themselves. Health practitioners with a high index of suspicion, from the nature of lesions presented with by the patients, are sometimes able to identify IPV sufferers. However, these are often under-reported and consequently poorly managed. The aim of this article is to motivate for a primary preventive approach to addressing the problem of IPV in Botswana.

## Global burden of intimate partner violence

Intimate partner violence is a pervasive social problem with about one in three women having experienced it once in their lifetime.^6^ A systematic review and meta-regression of 141 studies from 81 countries in 2010 estimated the global lifetime prevalence of IPV as 30% (95% confidence interval [CI] 27.8 to 32.2).^7^ However, this estimate excluded emotional and psychological intimate violence, which though not as readily substantiated is as traumatising and dehumanising as physical IPV.

The World Health Organization (WHO) in a review of data on IPV between 1982 and 2011 estimated that as many as 38% of homicidal deaths of women globally resulted from murders committed by their intimate partners.^6^. Intimate partner violence is thus the leading cause of female homicide globally, in South Africa and probably in most other countries in sub-Saharan Africa.^6,8,9^

Apart from injuries or death, victims of IPV are more likely to develop medical conditions like asthma, bladder and kidney infections, circulatory conditions, cardiovascular disease, fibromyalgia, irritable bowel syndrome, chronic pain syndromes, central nervous system disorders, gastrointestinal disorders, joint disease, migraines and headaches.^3,10,11^ Most IPV victims present with these medical conditions and are only detected to be IPV sufferers when the attending health practitioner has a holistic approach and a thorough clinical consultation. Thus, IPV causes increased health care utilisation with the attendant costs of managing the associated medical conditions.^3,10^

Women who experience physical, emotional or sexual IPV are also more likely to get infected with HIV.^12,13^ This is even more likely in an environment with a high HIV prevalence like Botswana. Coercive male controlling behaviour which is common in Botswana, especially when associated with physical and emotional IPV, has been associated with HIV infection.^8,12,14^ They are also more likely to have other sexually transmitted infections, unintended or unwanted pregnancies and negative pregnancy outcomes like preterm deliveries, low birth weight babies and perinatal deaths.^10,11^

Psychological disorders associated with IPV include anxiety, depression, post-traumatic stress disorder, antisocial behaviour, deliberate self-harm and suicide in females, low self-esteem, inability to trust others especially in intimate relationships, emotional detachment, sleep disturbances and flashbacks.^10^

Victims of IPV are also more likely to abuse psycho-active substances such as tobacco, alcohol and other illicit drugs as a maladaptive way of coping with stress. They may have multiple sexual partners, engage in high-risk sexual activities and transactional sex.^10^

Although data on IPV are not available in all countries, it appears that IPV is present in all countries globally. The intensity and extent may however vary according to different communities and cultures.

## Burden of intimate partner violence in Botswana

Although data on actual prevalence of IPV in Botswana are scanty, unpublished reports indicate a high prevalence of IPV in Botswana, with extreme homicidal cases locally referred to as passion killing. Passion killing is the common parlance for the murder of an individual (mostly female) by the intimate partner. The partner may be a current or estranged lover or even in some instances a man whose offer of a relationship was rejected by the victim.

A 2012 self-reported survey of 1229 (639 females and 590 males) citizens of Botswana aged 15 years and above, from remote settlements, rural and urban areas of Botswana, reported a lifetime prevalence of IPV of 67% among the women.^15^ More than one-quarter of the women interviewed admitted that they experienced some form of IPV within 12 months prior to the survey, while close to half of the men interviewed admitted perpetrating IPV.^15^

The most common type of IPV reportedly experienced by women interviewed was emotional.^14,15^ Emotional IPV could be in the form of verbal abuse, derogatory comments or actions, or by deprivation of physical, financial, psychological or material needs of the victim. Women aged 18–44 years were the most affected by IPV, and men aged 18–29 years perpetrated most of the IPV.^14,15^ A role for transgenerational relationships in IPV was not demonstrated in the survey conducted in Botswana.

In Botswana, educated and employed women appeared to experience more IPV compared with their unemployed and less educated counterparts, but it may be argued that educated and employed women in Botswana may be less tolerant of IPV and more likely to report their experience. Similarly, men with higher educational status admitted to perpetrating IPV more than men with lower levels of education.^14^ Here again, the role of lack of assertiveness as a result of economic dependence was not demonstrated in the Botswana context.

As was found in other southern African countries, cultural beliefs about the unquestionable rights of men to have sex on demand in relationships, and acceptance of partner beating as permissible, played a major role in the prevalence of IPV in Botswana.^14^

Childhood experience of abuse or violence seems to play an important role in later experience or perpetration of IPV in Botswana. Most (88%) of the women who had experienced IPV and about two-thirds of the men who admitted perpetrating IPV reported being abused as children. About half of these women had also witnessed their mothers experiencing IPV. A quarter of the men who admitted perpetrating IPV had witnessed their mothers experiencing IPV.^8,14,15^

## Drivers of intimate partner violence in Botswana

The key factors found to drive IPV in Botswana are gender attitude, alcohol and other substance abuse, and relationship conflicts.^8,13,16^

### Gender attitude

The notion of male dominance and female subservience is common and accepted by many communities in Botswana. Some form of discipline, physical or psychological, inflicted on their female partners is commonly culturally accepted as the right or prerogative of men in relationships. Most communities will only frown at the excessive measures used by men, such as that which results in profound injury or death of the partner. This gender attitude is promoted from childhood where the male child is accepted to be more aggressive and dominant while the girl child is encouraged to be more domestic, subservient and tolerant.^14,17,18^

### Alcohol and other substance abuse

Alcohol in both regulated and locally brewed forms are consumed by many youths in Botswana. Many also abuse other psycho-active substances, the most common of which is cannabis, locally known as dagga. About 25% of men who reported perpetrating IPV also admitted having taken alcohol and other intoxicating substances within 12 months prior to the survey.^15,16^

### Relationship conflicts

Suspicion of infidelity appears to contribute to the experience of IPV in Botswana. Half of the women who reported experiencing IPV within 12 months prior to the survey felt that their partners were having other affairs and cheating them, and it may be inferred that their approach to the relationship conflict may have played a role in their experience of IPV.^12,14,15^

## Current strategies used to address intimate partner violence in Botswana

### Public education

Various forms of educational materials, mainly in print media, are used to convey messages discouraging IPV. Once a while, non-governmental organisations promote walk-throughs in the community to draw attention to the evils of IPV.

### Customary courts (*kgotla*)

These courts provide jurisprudence over domestic issues and are a recourse for victims of IPV. The courts are presided over by the chief (*kgosi*) of the community. Usually, the relatives of the IPV victim report the cases when the victim has suffered significant injury from the perpetrator. The perpetrator is then penalised with corporal punishment such as strokes of cane and the case is usually concluded. The impact of these courts as a deterrent to IPV perpetration is very doubtful.

### Office of the district commissioner

This is the office with the statutory right for the legal wedding of couples in Botswana. It also serves as an arbitration centre where conflicts between partners may be resolved through the mediation of the district commissioner.

### The social works office

Patients identified by health workers as victims of IPV are usually referred for counselling by social workers. The counselling also includes negotiations for the safety of the victims and arrangements for alternative homes where appropriate. These social workers are based at the clinics, the district and the referral hospitals from where they conduct visits to homes in the community as needed. They receive patients with IPV only when they are referred to them by the attending health practitioners. They are often challenged by transport and other logistic constraints and apart from arranging safe shelters when requested by the victim, they are unable to provide conclusive assistance in most instances.

### The police department

Self-reports of IPV and referrals from the social works department are attended to by the police. Perpetrators of IPV are often arrested and charged for assault occasioning bodily harm when the victim or relatives of the victim are willing to press charges.

### Non-governmental organisations

These organisations handle self-referrals of IPV victims and intervene with counselling, arrangements for safety and support for litigation of the perpetrator. They are the main promoters of community education and motivation against IPV.

The focus of interventions against IPV in Botswana currently consists of actions taken, usually after the IPV has been going on for considerable periods. Community education and mobilisation against IPV are sporadic and often prompted by reports of severe violence or death – passion killing – of a victim. The mainly tertiary approach to addressing IPV does not appear to have had a significant impact in reducing the burden of IPV in Botswana.

## Rationale for a primary preventive approach to address intimate partner violence in Botswana

The association of IPV experience and perpetration with childhood abuse suggests that victims of such childhood abuse could be a focus of interventions to prevent IPV developing later.^19,20,21^

Interventions aimed at the male dominant and female subservient gender attitude in the social mores of the communities may also help to reduce IPV. These interventions could help reduce the community tolerance of IPV by highlighting IPV as a social ill rather than as a private domestic issue.^20,21,22^

Interventions addressing alcohol and other substance abuse may also help reduce IPV fostered by intoxication.^20,21,22^

Context-specific conflict resolution structures and promotion of healthy interpersonal communication may help to create avenues for resolution of relationship conflicts and prevent IPV.^21^

## Challenges for a primary preventive approach to intimate partner violence in Botswana

Intimate partner violence is a complex multifactorial problem and a multifaceted approach to the prevention would be expensive. The impact of the interventions may only be evident after a considerable time period. This may be a challenge for the sourcing of donor funds to support the programmes. The Government of Botswana has not shown targeted budget towards the prevention of IPV but provides ad hoc support for non-governmental organisations’ activities against perpetration of IPV. Most donor organisations would expect documented evidence of impact within restricted time of disbursement of the funds.

Most IPV preventive programmes have not been rigorously evaluated. The few that have been were mainly undertaken in developed countries. Thus, there are very few similar, contextually related programmes to benchmark against.^20,21,23,24^ ‘Stepping Stones’, a participatory HIV prevention programme that promotes gender-equitable sexual behaviour was used in a community-based randomised control study in South Africa. It demonstrated substantial reduction in self-reported perpetration of IPV by the intervention arm participants.^22,25^ Earlier, another community-based randomised study, also in rural South Africa, which combined microfinance and gender-equitable behavioural training, similarly demonstrated substantial reduction in IPV in the intervention community.^13,26^

To be effective, the preventive approach has to be well planned, socially acceptable, systematically implemented and thoroughly evaluated. There will also be a need for commitment of stakeholders, dedicated human and material resources, and adequate financial support.^20,21^

## Designing evidence-based preventive programmes for Botswana

The programme should be theory driven: the programme should aim to intervene, guided by the theoretic basis of IPV.

Three theories have been proposed for IPV development^17,19,20,21,27,28^:

- social learning theory, which suggests that IPV develops as a result of experiences learnt in childhood and the role of influential models in the life of the developing child
- feminist theory, which suggests that IPV develops because of deep cultural beliefs about the subservient nature of women and the dominant, patriarchal role of men
- attachment theory, which suggests that insecure relationship experienced with primary caregivers in early childhood results in low self-esteem and insecurity in consequent intimate relationships.

Programmes designed on the basis of the social learning and attachment theories could target children identified with childhood abusive experience for concerted interventions such as cognitive behaviour therapy (CBT). This may help to prevent them from experiencing or perpetrating IPV in later life. However, CBT was not found to consistently prevent recidivism among adults who had experienced or perpetrated IPV.^18,29^ Also, identifying such vulnerable children and providing them with the intervention could be challenging.

Addressing the feminist theory would need programmes targeting key leaders and influential stakeholders in the communities for continuous reorientation of their perception of gender roles. Workshops, seminars, theatrical performances and other means may be used to gradually change the paradigm of people in the community.

The programmes should be comprehensive in scope: different driving factors for behaviours leading to IPV in the Botswana context should be addressed concurrently. Aspects of the programme could focus on training healthy ways of communication and conflict resolution in relationships. Other aspects could dwell on motivating behavioural change on alcohol or other substance abuse at individual levels and developing communally acceptable strategies to discourage excessive alcohol intake.^13,18,21,26^

Ideally, the programmes should focus on preadolescents and adolescents, and they should be implemented in youth-friendly ways as this would promote better cooperation and participation by these youths.

The programmes should have a variety of presentation modalities. As much as possible, they should be interactive, engaging the youths themselves in lively hands-on learning sessions. Peer influence should be used positively by having fellow youths as guest speakers in discussions, symposia or seminars. Role plays, theatrical performances and peer education may also be effective ways of presenting the programmes. Involving the youths themselves in the programme delivery and the use of lively interactive formats would be more acceptable to the preadolescent and adolescent age groups targeted for the programme. The interventions should encourage healthy behaviours in both intimate and regular, non-intimate relationships such as with their teachers and neighbours^21,23,30^

Programmes targeting community leaders may be enhanced by the use of video shows, highlighting the effects of IPV. Small group participatory workshops may also be efficient ways of challenging existing beliefs and encouraging collective action against IPV. There may be the need for adequate education and preparation of community leaders to obtain their acceptance and support for the programme. Every effort should be made to ensure that the programmes are sensitive to and respect the sociocultural norms of the community. This can be performed by avoiding direct criticism of the sociocultural mores of the community and facilitating their own self-appraisal of the consequences of their belief paradigm in ways similar to what is used in psychosocial counselling.^21,23,29,31,32^

Intimate partner violence involves behaviours and beliefs that are deeply rooted. Many are encouraged by cultural mores that have existed for generations in the community. Programmes that would influence changes in belief paradigms and eventually motivate healthy relationship behaviours need to be delivered in sufficient time and consistently for considerable duration. There are no empirical data to support optimal timing of the intervention delivery, and they may be context specific depending on the prevalence of driving factors for IPV in the community. It is expedient to provide multiple sessions of programme interventions and continuous reinforcement over 2 years or more before expecting a significant impact on IPV.^11,21,28,33^

The quality of the interaction with the participants during the delivery of the interventions is crucial to the success of the programmes. Thus, intervention leaders should be well trained and should adhere to the intervention protocols. They should be experienced in youth issues and have good knowledge of IPV. They should also be able to handle behavioural issues that may arise during the delivery of the interventions.^20,21,22,34^

Programmes addressing alcohol and other substance abuse as driving factors for IPV may consider provision of safer alternative recreational outlets for youths. Youths are usually energetic and tend to be restless when idle. Thus, occupying them with avenues to expend their energies on safer activities may help reduce their resorting to alcohol. Youths and adolescents are also more flexible and open to trying out new things. This should make this age group more amenable to change, the bastion for a primary preventive approach to addressing the problem of IPV.^27^ A programme on the prevention of IPV may also be considered for inclusion in the primary and secondary education curriculum in Botswana. Increasing access to and encouraging participation in various sports like table tennis, chess, badminton, squash, football and swimming could engage the youths in safer activities and help reduce the recourse by youths to alcohol when idle. Inter-ward or inter-district sporting competitions may also be used to engage the interest of the youths in these sports, which may offer safer outlets for them to expend their energy.^17,22,26,34^

Opportunities for mass education of married men and women, and those about to get married, on the value of trust in relationships, stressing the avoidance of prying into the privacy of their partners, should be explored. This is a form of stalking and may result in relationship conflicts and IPV.

The goals and objectives of the programmes should be well outlined from the onset and should be feasible and measurable on achievement. Financial support for the programmes may require prioritisation by the government. This could be motivated for with appropriate, well-articulated proposals. Donor agencies may also be approached for financial leverage. Community-based randomised control trials are the best designs for demonstrating the impact of the programmes.^20,21^

## Conclusion

To address the problem of IPV in Botswana, a shift in preventive modality from the present tertiary to a primary approach may yield more significant impact. However, a contextually relevant primary preventive strategy requires systematic, coordinated and sustained implementation of programmes with a comprehensive scope and commitment from all stakeholders. With proper planning and adequate financing, the primary preventive approach portends better prospects of significantly reducing the burden of IPV in Botswana.
